# Supplementary material for: Machine learning analyses of antibody somatic mutations predict immunoglobulin light chain toxicity
Source: Nat Commun. 2021 Jun 10;12:3532. doi: 10.1038/s41467-021-23880-9 (PMC8192768; doi:10.1038/s41467-021-23880-9)
Supplement: Supplementary file 1 — Supplementary information. [file 41467_2021_23880_MOESM1_ESM.pdf]

SUPPLEMENTARY FIGURES

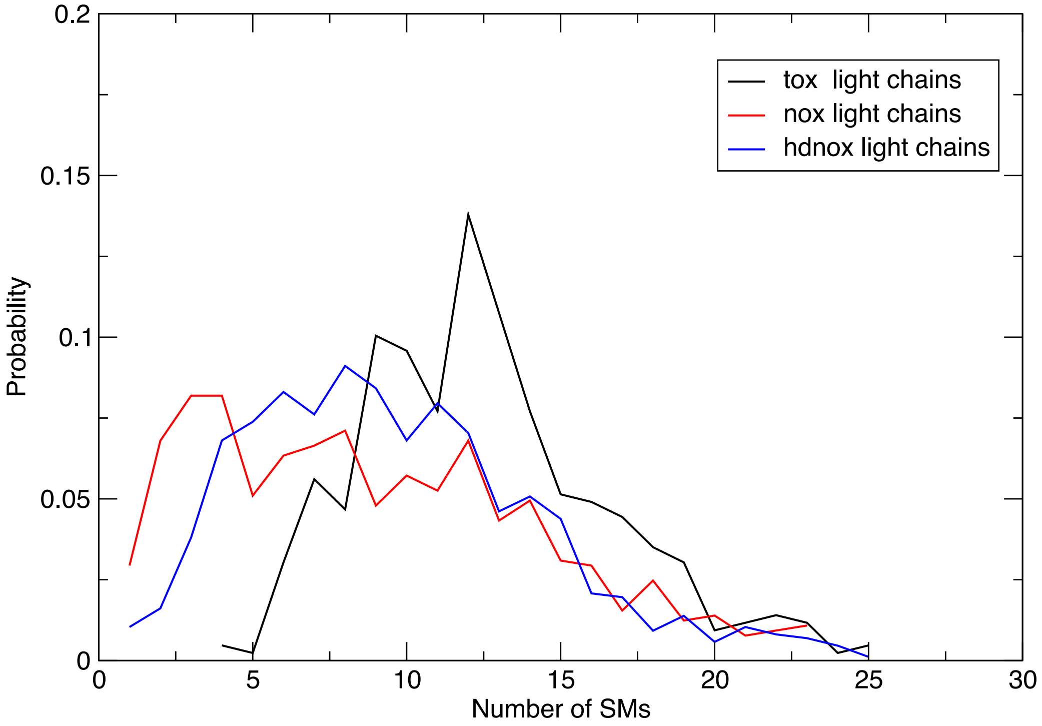

Supplementary Figure. 1 | Probability distribution of somatic mutations in toxic and non-toxic light chain sequences. The black line represent the probability distributions of somatic mutations (PDSM) in toxic light chains (tox) (average number of mutations equal to 12.5), the red line represent the PDSM of non-toxic sequences (nox) used in the training of the machine learning algorithm (average number of mutations equal to 8.8), while the blue line represent the PDSM computed randomly selecting 1000 lambda LCs from a healthy donor[1] (hdnox) (average number of mutations equal to 9.6). The difference in average number of somatic mutations in the two set of non-toxic LC sequences is not statistically significant ($p = 0.1$), while there is a statistically significant difference between the toxic sequences and both non-toxic dataset ($p < 10e-29$). Statistical analysis were performed using Student *t* test.

a

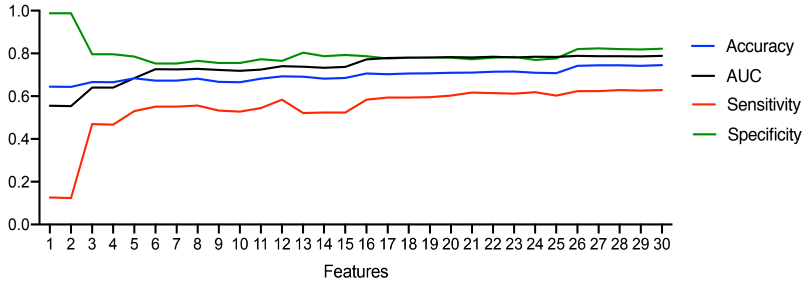

b

	Features
1	49A
2	49A.I49AX116I
3	49A.I49AX116I.I65XX107I
4	49A.I49AX116I.I65XX107I.I4XA49I
5	49A.I49AX116I.I65XX107I.I4XA49I.I49XX99I
6	49A.I49AX116I.I65XX107I.I4XA49I.I49XX99I.I52XX108I
7	49A.I49AX116I.I65XX107I.I4XA49I.I49XX99I.I52XX108I.I49AX99I
8	49A.I49AX116I.I65XX107I.I4XA49I.I49XX99I.I52XX108I.I49AX99I.107X
9	49A.I49AX116I.I65XX107I.I4XA49I.I49XX99I.I52XX108I.I49AX99I.107X._104XX108_
10	49A.I49AX116I.I65XX107I.I4XA49I.I49XX99I.I52XX108I.I49AX99I.107X._104XX108_.I3XA49I
11	49A.I49AX116I.I65XX107I.I4XA49I.I49XX99I.I52XX108I.I49AX99I.107X._104XX108_.I3XA49I.106X
12	49A.I49AX116I.I65XX107I.I4XA49I.I49XX99I.I52XX108I.I49AX99I.107X._104XX108_.I3XA49I.106X._44XX98_
13	49A.I49AX116I.I65XX107I.I4XA49I.I49XX99I.I52XX108I.I49AX99I.107X._104XX108_.I3XA49I.106X._44XX98_._52XX65_
14	49A.I49AX116I.I65XX107I.I4XA49I.I49XX99I.I52XX108I.I49AX99I.107X._104XX108_.I3XA49I.106X._44XX98_._52XX65_._44XX99_
15	49A.I49AX116I.I65XX107I.I4XA49I.I49XX99I.I52XX108I.I49AX99I.107X._104XX108_.I3XA49I.106X._44XX98_._52XX65_._44XX99_.I66XX107I
16	49A.I49AX116I.I65XX107I.I4XA49I.I49XX99I.I52XX108I.I49AX99I.107X._104XX108_.I3XA49I.106X._44XX98_._52XX65_._44XX99_.I66XX107I._54XX58_
17	49A.I49AX116I.I65XX107I.I4XA49I.I49XX99I.I52XX108I.I49AX99I.107X._104XX108_.I3XA49I.106X._44XX98_._52XX65_._44XX99_.I66XX107I._54XX58_._1XH108_
18	49A.I49AX116I.I65XX107I.I4XA49I.I49XX99I.I52XX108I.I49AX99I.107X._104XX108_.I3XA49I.106X._44XX98_._52XX65_._44XX99_.I66XX107I._54XX58_._1XH108_.I49XX116I
19	49A.I49AX116I.I65XX107I.I4XA49I.I49XX99I.I52XX108I.I49AX99I.107X._104XX108_.I3XA49I.106X._44XX98_._52XX65_._44XX99_.I66XX107I._54XX58_._1XH108_.I49XX116I.108H
20	49A.I49AX116I.I65XX107I.I4XA49I.I49XX99I.I52XX108I.I49AX99I.107X._104XX108_.I3XA49I.106X._44XX98_._52XX65_._44XX99_.I66XX107I._54XX58_._1XH108_.I49XX116I.108H._44HX96_
21	49A.I49AX116I.I65XX107I.I4XA49I.I49XX99I.I52XX108I.I49AX99I.107X._104XX108_.I3XA49I.106X._44XX98_._52XX65_._44XX99_.I66XX107I._54XX58_._1XH108_.I49XX116I.108H._44HX96_._44XX97_
22	49A.I49AX116I.I65XX107I.I4XA49I.I49XX99I.I52XX108I.I49AX99I.107X._104XX108_.I3XA49I.106X._44XX98_._52XX65_._44XX99_.I66XX107I._54XX58_._1XH108_.I49XX116I.108H._44HX96_._44XX97_.78X
23	49A.I49AX116I.I65XX107I.I4XA49I.I49XX99I.I52XX108I.I49AX99I.107X._104XX108_.I3XA49I.106X._44XX98_._52XX65_._44XX99_.I66XX107I._54XX58_._1XH108_.I49XX116I.108H._44HX96_._44XX97_.78X.I52XX116I
24	49A.I49AX116I.I65XX107I.I4XA49I.I49XX99I.I52XX108I.I49AX99I.107X._104XX108_.I3XA49I.106X._44XX98_._52XX65_._44XX99_.I66XX107I._54XX58_._1XH108_.I49XX116I.108H._44HX96_._44XX97_.78X.I52XX116I.106N
25	49A.I49AX116I.I65XX107I.I4XA49I.I49XX99I.I52XX108I.I49AX99I.107X._104XX108_.I3XA49I.106X._44XX98_._52XX65_._44XX99_.I66XX107I._54XX58_._1XH108_.I49XX116I.108H._44HX96_._44XX97_.78X.I52XX116I.106N._42XX52_
26	49A.I49AX116I.I65XX107I.I4XA49I.I49XX99I.I52XX108I.I49AX99I.107X._104XX108_.I3XA49I.106X._44XX98_._52XX65_._44XX99_.I66XX107I._54XX58_._1XH108_.I49XX116I.108H._44HX96_._44XX97_.78X.I52XX116I.106N._42XX52_._56XX59_
27	49A.I49AX116I.I65XX107I.I4XA49I.I49XX99I.I52XX108I.I49AX99I.107X._104XX108_.I3XA49I.106X._44XX98_._52XX65_._44XX99_.I66XX107I._54XX58_._1XH108_.I49XX116I.108H._44HX96_._44XX97_.78X.I52XX116I.106N._42XX52_._56XX59_.49X
28	49A.I49AX116I.I65XX107I.I4XA49I.I49XX99I.I52XX108I.I49AX99I.107X._104XX108_.I3XA49I.106X._44XX98_._52XX65_._44XX99_.I66XX107I._54XX58_._1XH108_.I49XX116I.108H._44HX96_._44XX97_.78X.I52XX116I.106N._42XX52_._56XX59_.49X.44X
29	49A.I49AX116I.I65XX107I.I4XA49I.I49XX99I.I52XX108I.I49AX99I.107X._104XX108_.I3XA49I.106X._44XX98_._52XX65_._44XX99_.I66XX107I._54XX58_._1XH108_.I49XX116I.108H._44HX96_._44XX97_.78X.I52XX116I.106N._42XX52_._56XX59_.49X.44X.52X
30	49A.I49AX116I.I65XX107I.I4XA49I.I49XX99I.I52XX108I.I49AX99I.107X._104XX108_.I3XA49I.106X._44XX98_._52XX65_._44XX99_.I66XX107I._54XX58_._1XH108_.I49XX116I.108H._44HX96_._44XX97_.78X.I52XX116I.106N._42XX52_._56XX59_.49X.44X.52X.44H

Supplementary Figure. 2 | Quantitative analysis of the importance of features identified by the feature selection technique training 30 different classifiers with a 10-fold cross validation, adding one by one the 10 most important features of each feature family according to information gain. **a,** the contribution of each feature in the achieved AUC, Accuracy, Sensitivity and Specificity, and **b**, the specific features added. The results of these analysis are reported in Supplementary Table 9.

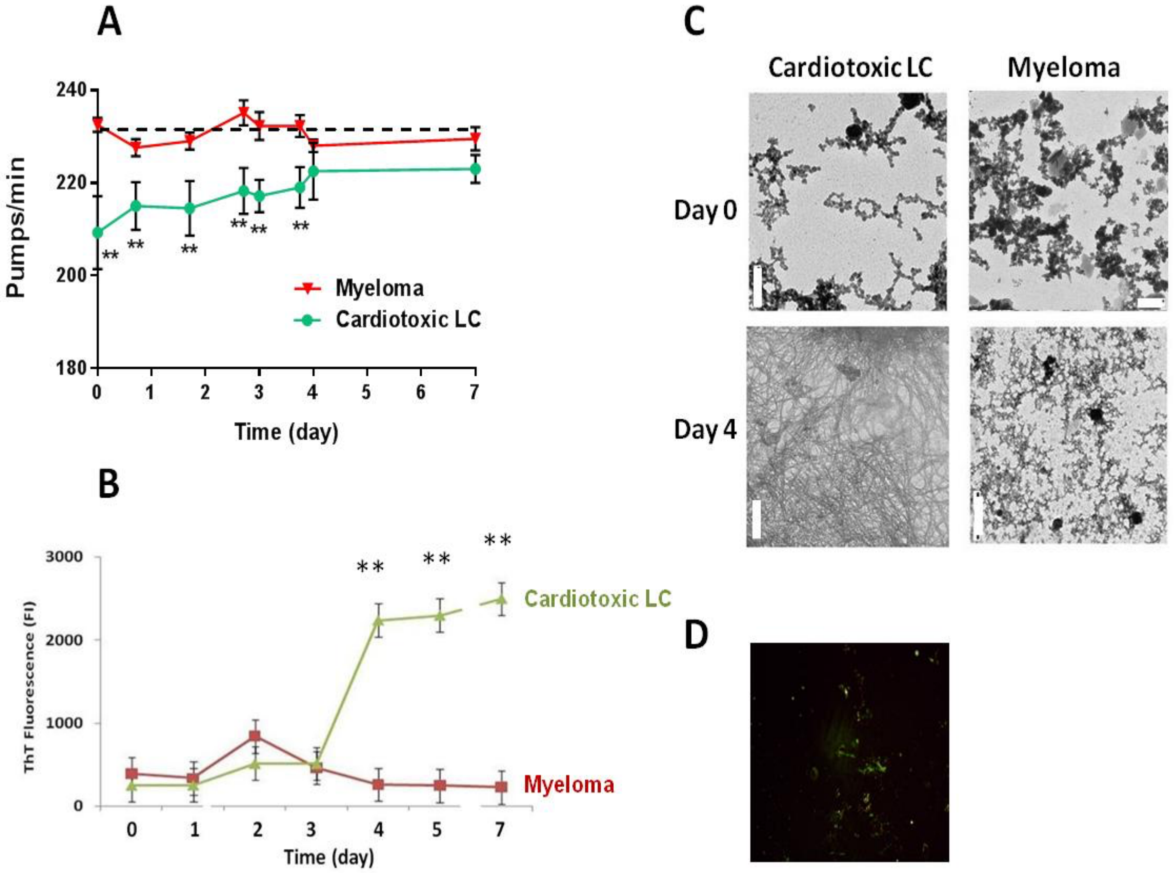

Supplementary Figure 3 | Amyloidogenic cardiotoxic LC and nonamyloidogenic myeloma LC (Myeloma) were suspended at 20 μM in 10 mM sodium acetate, boric acid, and sodium citrate buffer containing 150 mM NaCl, pH 2.0, and incubated at 67°C. Buffer alone (vehicle) was incubated as control. (**A**) The toxicity of LCs was determined before and different times after incubation by administering them to N2 *C. elegans* (100 worms/100 μl) at 100 μg/ml for 2 h. Control worms fed vehicle alone. The pharyngeal pumping rate was scored 24 h after the administration. ** $p<0.001$ *vs* vehicle (dotted line) and myeloma according to one-way Anova and Bonferroni's *post hoc* test. (**B**) Fibrillogenesis was monitored before and different times after incubation by determining the fluorescence intensity of ThT. ** $p<0.001$ *vs* myeloma at the same time point, Student's t-test. (**C**) TEM analysis was performed before (Day 0) and 4 days after incubation (Day 4) by negatively stained samples with 2% uranyl acetate. Scale bar=0.2 μm. (**D**) Cardiotoxic LC incubated for 4 days were stained with Congo Red and observed at a polarized microscopy.

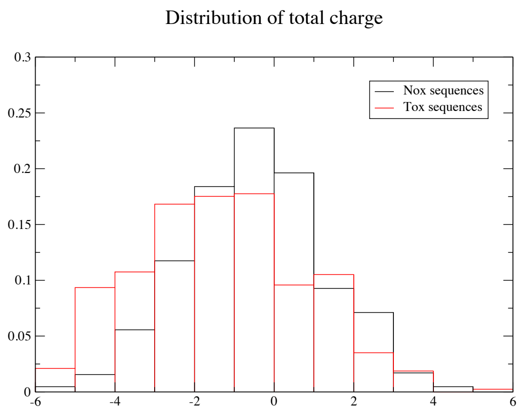

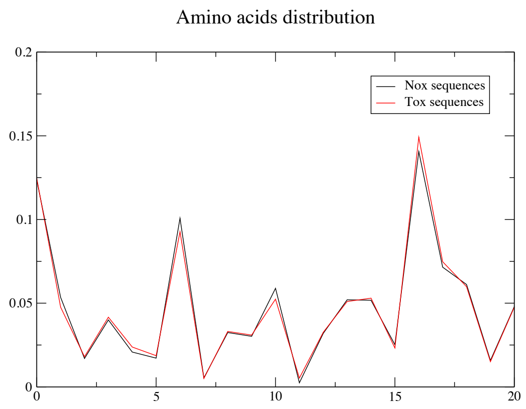

Supplementary Figure 4 | Comparison of charge and amino acids distributions in *nox* and *tox* sequences.

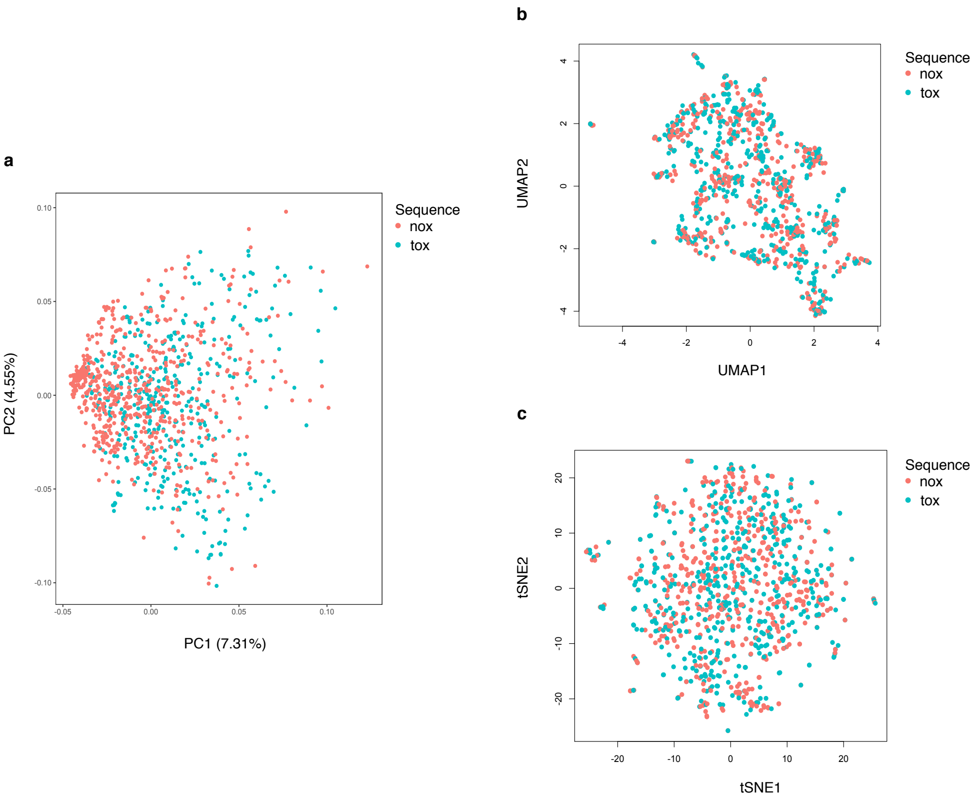

Supplementary Figure 5 | a, Principal component analysis (PCA) on *tox* and *nox* sequences expressed as binary vectors of dimension 125x21, where for each amino acid we used a one-hot encoding vector of dimension 21 (20 amino acids and possible insertions). **b**, t-SNE and **c**, UMAP analyses.
