# Supplementary material for: Machine learning analyses of antibody somatic mutations predict immunoglobulin light chain toxicity
Source: Nat Commun. 2021 Jun 10;12:3532. doi: 10.1038/s41467-021-23880-9 (PMC8192768; doi:10.1038/s41467-021-23880-9)
Supplement: Supplementary file 2 — Reporting summary. [file 41467_2021_23880_MOESM2_ESM.pdf]

nature research

Corresponding author(s): Andrea Cavalli

Last updated by author(s): 5/18/2021

Reporting Summary

Nature Research wishes to improve the reproducibility of the work that we publish. This form provides structure for consistency and transparency in reporting. For further information on Nature Research policies, see our Editorial Policies and the Editorial Policy Checklist.

Statistics

For all statistical analyses, confirm that the following items are present in the figure legend, table legend, main text, or Methods section.

n/a	Confirmed	
☐	☒	The exact sample size (*n*) for each experimental group/condition, given as a discrete number and unit of measurement
☐	☒	A statement on whether measurements were taken from distinct samples or whether the same sample was measured repeatedly
☒	☐	The statistical test(s) used AND whether they are one- or two-sided<br>*Only common tests should be described solely by name; describe more complex techniques in the Methods section.*
☒	☐	A description of all covariates tested
☐	☒	A description of any assumptions or corrections, such as tests of normality and adjustment for multiple comparisons
☐	☒	A full description of the statistical parameters including central tendency (e.g. means) or other basic estimates (e.g. regression coefficient) AND variation (e.g. standard deviation) or associated estimates of uncertainty (e.g. confidence intervals)
☐	☒	For null hypothesis testing, the test statistic (e.g. F, t, r) with confidence intervals, effect sizes, degrees of freedom and P value noted<br>*Give P values as exact values whenever suitable.*
☒	☐	For Bayesian analysis, information on the choice of priors and Markov chain Monte Carlo settings
☒	☐	For hierarchical and complex designs, identification of the appropriate level for tests and full reporting of outcomes
☐	☒	Estimates of effect sizes (e.g. Cohen's d, Pearson's r), indicating how they were calculated

Our web collection on statistics for biologists contains articles on many of the points above.

Software and code

Policy information about availability of computer code

Data collection	No software was used to collect data for this study.
Data analysis	For data analysis we used R version 3.5.1 and GraphPad Prism 8.2.1. Weka 3.8.1 was used for machine learning algorithms. Code is available at code: https://github.com/mauragarofalo/LICTOR/code.

For manuscripts utilizing custom algorithms or software that are central to the research but not yet described in published literature, software must be made available to editors and reviewers. We strongly encourage code deposition in a community repository (e.g. GitHub). See the Nature Research guidelines for submitting code & software for further information.

Data

Policy information about availability of data

All manuscripts must include a data availability statement. This statement should provide the following information, where applicable:

- Accession codes, unique identifiers, or web links for publicly available datasets
- A list of figures that have associated raw data
- A description of any restrictions on data availability

All nox and tox sequences used are available in the GitHub repositiry: https://github.com/mauragarofalo/LICTOR/data.
Amyloid Light-chain Database: http://albase.bumc.bu.edu.

Field-specific reporting

Please select the one below that is the best fit for your research. If you are not sure, read the appropriate sections before making your selection.

- [x] Life sciences
- [ ] Behavioural & social sciences
- [ ] Ecological, evolutionary & environmental sciences

For a reference copy of the document with all sections, see nature.com/documents/nr-reporting-summary-flat.pdf

Life sciences study design

All studies must disclose on these points even when the disclosure is negative.

Sample size	Each experiment aimed at determining the proteotoxicity of LCs were performed using 100 worms per experimental group. Experiments were repeated at least two times. The choice of these numbers was based on methods described on http://www.wormbook.org/toc_wormmethods.html and validated in our previous manuscripts on C. elegans and LC toxicity (Diomede et al., 2014 and 2017).
Data exclusions	No data were excluded from analysis.
Replication	Experiments aimed at determining the proteotoxicity of LCs were repeated two times according to http://www.wormbook.org/toc_wormmethods.html and our previous manuscripts on C. elegans and LC toxicity (Diomede et al., 2014 and 2017). All attempts at replication were successful.
Randomization	C.elegans were randomly divided in groups.
Blinding	C. elegans were treated by an investigator which marked samples by numbers: a second investigator determined the pharyngeal pumping in blind.

Reporting for specific materials, systems and methods

We require information from authors about some types of materials, experimental systems and methods used in many studies. Here, indicate whether each material, system or method listed is relevant to your study. If you are not sure if a list item applies to your research, read the appropriate section before selecting a response.

Materials & experimental systems

n/a	Involved in the study	
[x]	[ ]	Antibodies
[ ]	[x]	Eukaryotic cell lines
[x]	[ ]	Palaeontology and archaeology
[ ]	[x]	Animals and other organisms
[x]	[ ]	Human research participants
[x]	[ ]	Clinical data
[x]	[ ]	Dual use research of concern

Methods

n/a	Involved in the study	
[x]	[ ]	ChIP-seq
[x]	[ ]	Flow cytometry
[x]	[ ]	MRI-based neuroimaging

Eukaryotic cell lines

Policy information about cell lines

Cell line source(s)	Expi293F (Thermo Fisher Scientific) .
Authentication	Not authenticated after purchase
Mycoplasma contamination	In situ analysis never detected Mycoplasma infection.
Commonly misidentified lines (See ICLAC register)	No commonly misidentified cell line have been used in this study.

Animals and other organisms

Policy information about studies involving animals; ARRIVE guidelines recommended for reporting animal research

Laboratory animals	Bristol N2 Caenorhabditis elegans, hermaphrodite, L3-L4 larval stage
Wild animals	The study did not involve wild animals
Field-collected samples	The study did not involve samples collected from the field

Ethics oversight	No ethical approval or guidelines were required to use C. elegans

Note that full information on the approval of the study protocol must also be provided in the manuscript.
